# Pelvic inflammatory disease and causative pathogens in older women in a medical center in eastern Taiwan: A retrospective cross-sectional study

**DOI:** 10.1371/journal.pone.0257627

**Published:** 2021-09-20

**Authors:** Pei-Chen Chen, Pei-Chen Li, Dah-Ching Ding

**Affiliations:** 1 Department of Obstetrics and Gynecology, Hualien Tzu Chi Hospital, Buddhist Tzu Chi Medical Foundation, Tzu Chi University, Hualien, Taiwan; 2 Institute of Medical Sciences, Tzu Chi University, Hualien, Taiwan; University Medical Center Utrecht, NETHERLANDS

## Abstract

**Objectives:**

Most research into the management of pelvic inflammatory disease (PID) is in younger women and focuses on sexually transmitted pathogens such as *N*. *gonorrhoeae* or *C*. *trachomatis*. Non-sexually transmitted bacterial pathogens and PID in older women are rarely examined. The objective of this study is to explore cervical culture pathogens in women of different age groups in a medical center in eastern Taiwan.

**Methods:**

We enrolled patients whose medical records were diagnosed with PID (ICD-9-CM 614.0 [N70.01–03], 614.1[N70.11–13], 614.9 [N73.5, N73.9]) at our hospital from October 2014 to March 2020. Patients were divided into three groups according to age: the age <25 years, age 25–44 years, and the ≥ 45 years group. Chi-square test, ANOVA and logistic regression were used for statistical analysis. In subgroup analysis, endocervical pathogens were further stratified into vaginal, respiratory, enteric, skin, oral, and other.

**Results:**

A total of 96 patients were included in the study. There were 31 patients in the age ≥ 45 years group, 52 patients in the age 25–44 years group, and 13 patients in the age <25 years group. Vagina and enteric pathogens were the most common pathogens among all groups. The isolated respiratory and other pathogens were more in the age ≥ 45 years group than in the other two groups. *Prevotella bivia* was more common in the age <25 years and 25–44 years groups.

**Conclusions:**

This may be due to different pathogeneses of PID in the age ≥ 45 years patients. Our study can be used as a reference for antibiotic choice of non-sexually transmitted PID and to prevent long-term sequelae of PID.

## Introduction

Pelvic inflammatory disease (PID) is an infectious disease of the pelvic cavity, including the upper genital tract. Patients typically present with pain, abnormal discharge, lower abdominal pain, and fever [1]. Women with PID are at higher risk of chronic pelvic pain, ectopic pregnancy, infertility, and internal pelvic scarring [2, 3]. PID is associated with sexual activity, multiple sexual partners, young age, and previous PID [4]. Much of the research in PID has focused primarily on young, sexually active women. Among women aged 15–29 years, there are approximately 750,000 cases of PID each year in the US [5].

The most common pathogenesis of PID is the ascending infection of *N*. *gonorrhoeae* or *C*. *trachomatis* through sexual activity. The prevalence of PID associated with the above two pathogens is less than 50% [6, 7]. Other species of pathogens may play roles in causing PID. Additionally, vaginal microbiota and pathogens play a large role in PID and other infertility-associated problems [8]. In clinical situations, direct culture bacteria from the pelvic cavity is not available. Vaginal culture is an alternative way to obtain the information of PID causing pathogens. However, the relationship between bacterial vaginosis and PID remains controversial. A previous review summarized there were four categories of PID infectious agents, including cervical, bacterial vaginosis, respiratory, and enteric pathogens [9]. These pathogens may cause PID other than *N*. *gonorrhoeae* and *C*. *trachomatis* and show equal importance in PID treatment [10]. One study also showed a 5-fold decrease of PID caused by *C*. *Trachomatis* with age, so it is important to evaluate the other causes of PID in older women [11].

The management for PID follows the 2021 CDC (center for disease control and prevention) sexually transmitted disease (STD) guidelines [12]. Intravenous, intramuscular or oral antibiotics are the primary treatment. However, the guidelines focus on STD-related PID in young women. Study of pathogens of PID in older women is rare. Moreover, very few studies have been done in Taiwan or other countries examining the prevalence of non-sexually transmitted pathogens in PID, particularly among older women.

Therefore, in this study, we explore the cervical culture pathogens (except *N*. *gonorrhoeae* and *C*. *trachomatis*) in different age groups (<25, 25–44, and ≥45 years) of women in our institution in eastern Taiwan and identify other bacterial pathogen in PID for accurate antibiotic treatment in different ages of women.

## Materials and methods

### Data sources

We used an electronic medical history database in our hospital to retrieve the records of patients with PID over a 6-year period (2014–2020). The diagnostic codes were based on the International Classification of Diseases, Ninth and Tenth Revision, and Clinical Modification (ICD-9-CM and ICD-10-CM) during the study period. This study was approved by the Research Ethics Committee of Hualien Tzu Chi Hospital (IRB 108-201-B). The requirement for informed consent was waived due to minimal risk and approved by the Research Ethics Committee of Hualien Tzu Chi Hospital. We confirm that all methods were performed in accordance with the relevant guidelines and regulations.

### Study design and participants

This was a retrospective cross-sectional study with chart review. The inclusion criteria were patients’ medical records with the diagnosis of PID in our hospital. The exclusion criteria were patients’ medical records without cervical culture data or culture revealed *N*. *gonorrhoeae* and *C*. *trachomatis*. We used the ICD-9-CM [ICD-10-CM] codes for PID (614.0 [N70.01, N70.02, N70.03], 614.1 [N70,11, N70.12, N70.13], 614.9 [N73.5, N73.9]) to recruit patients from outpatient clinics and patients admitted to our hospital from October 2014 to March 2020.

Clinical diagnostic criteria for PID included lower abdominal pain, uterine or adnexal tenderness, cervical motion tenderness, and abnormal vaginal discharge [13].

Records for microbial culture for cases of PID were retrieved and analyzed.

### Outcome measures

Patients were divided into three groups according to age: <25 years, 25–44 years, and ≥45 years. The following data were collected: age, body mass index (BMI), education level, previous surgical history (gynecological or abdominal), birth history (normal spontaneous delivery [NSD] or cesarean delivery [C/S]), sexual history, number of sexual partner, smoking history, menopause, previous PID history, associated urinary tract infection (UTI), intrauterine device (IUD) placement, medical history (hypertension (HTN), diabetes mellitus (DM), or gastrointestinal problems), gynecologic structure problems (polyps or myoma), and pelvic organ prolapse.

In subgroup analysis, the endocervical pathogens were further stratified into vaginal (*Peptostreptococcus species*, *Bacteroides species*, *Atopobium species*, *Leptotrichia species*, *M*. *hominis*, *Ureaplasma urealyticum*, and *Clostridia species*), respiratory (*Haemophilus influenzae*, *Streptococcus pneumoniae*, *group A Streptococci*, and *Staphylococcus aureus*), enteric (*Escherichia coli*, *Bacteroides fragilis*, *group B streptococci*, and *Campylobacter species*), skin (Coagulase-negative staphylococci), oral (*Fusobacterium species*, *Streptococcus anginosus*, *β-streptococci group G*), and others (*Pantoea species*, *Pasteurella species*, *Shewanella putrefaciens*) [9].

### Statistical analyses

Statistical analysis was conducted with SPSS 25.0 (IBM, Armonk, NY, USA). The Chi-square test was used to compare categorical variables and ANOVA with the post-hoc Bonferroni test was used to compare continuous variables. Logistic regression was used to find the association between pathogens and demographics. A P-value < 0.05 was considered significant.

## Results

Ninety-six patients were included in the study: 31 patients in the age ≥45y group, 52 patients in the 25-44y group, and 13 patients in the <25 y group. Table 1 shows the characteristics of the patients. The number of vaginal deliveries, menopause, hypertension, and DM were significantly more common in the age ≥45 years group than in the other two groups. UTI was common in the age <25 years group. IUD use was common in the age 25–44 years group. There were no differences in BMI, education, previous PID, number of sexual partners, history of abdominal surgery, pelvic organ prolapse, or gynecological structure problems (myoma and polyps) among the three groups.

**Table 1:** Demographics (n = 96).

	<25 y/o (A)	25–44 y/o (B)	≧45 y/o (C)	Total	P-value	Post-hoc
**N**	13	52	31	96		
**Age**	20.54±2.15	35.17±6.04	55.29±10.94	39.69±14.10	<0.001*	A<B<C
**BMI**	21.97±5.11	25.86±6.72	26.83±5.64	25.65±6.32	0.075	
**BMI group**	-	-	-	-	0.368	
Normal	10(83.3%)	23(46.9%)	15(51.7%)	48(53.3%)		
Underweight	0(0.0%)	3(6.1%)	0(0.0%)	3(3.3%)		
Overweight	1(8.3%)	12(24.5%)	9(31.0%)	22(24.4%)		
Obese	1(8.3%)	11(22.4%)	5(17.2%)	17(18.9%)		
**Education**	-	-	-	-	0.305	
Junior high school or under	2(15.4%)	8(15.4%)	11(35.5%)	21(20.8%)		
Senior high school	8(61.5%)	23(44.2%)	9(29.0%)	43(42.6%)		
College or above	2(15.4%)	12(23.1%)	5(16.1%)	20(19.8%)		
Not mentioned	1(7.7%)	9(17.3%)	6(19.4%)	17(16.8%)		
**No. of NSD**	0.42±1.44	1.02±1.36	1.86±1.94	1.21±1.64	0.016*	A<C
**No. of C/S**	0.00±0.00	0.58±0.84	0.76±1.19	0.56±0.93	0.058	
**Delivery mode**	-	-	-	-	0.001*	
None	11(91.7%)	13(26.0%)	4(13.8%)	28(30.8%)		
NSD	1(8.3%)	18(36.0%)	15(51.7%)	34(37.4%)		
C/S	0(0.0%)	13(26.0%)	7(24.1%)	20(22.0%)		
Both	0(0.0%)	6(12.0%)	3(10.3%)	9(9.9%)		
**Menopause**	-	-	-	-	<0.001*	
No	13(100.0%)	49(94.2%)	12(38.7%)	74(77.1%)		
Yes	0(0.0%)	2(3.8%)	18(58.1%)	20(20.8%)		
Not mentioned	0(0.0%)	1(1.9%)	1(3.2%)	2(2.1%)		
**Previous PID**	-	-	-	-	0.812	
No	3(23.1%)	20(38.5%)	12(38.7%)	35(36.5%)		
Yes	4(30.8%)	16(30.8%)	8(25.8%)	28(29.2%)		
Not mentioned	6(46.2%)	16(30.8%)	11(35.5%)	33(34.4%)		
**Associated UTI**	-	-	-	-	0.039*	
No	7(53.8%)	36(69.2%)	18(58.1%)	61(63.5%)		
Yes	4(30.8%)	13(25.0%)	4(12.9%)	21(21.9%)		
Not mentioned	2(15.4%)	3(5.8%)	9(29.0%)	14(14.6%)		
**IUD use**	-	-	-	-	0.014*	
No	13(100.0%)	44(84.6%)	23(74.2%)	80(83.3%)		
Yes	0(0.0%)	5(9.6%)	0(0.0%)	5(5.2%)		
Not mentioned	0(0.0%)	3(5.8%)	8(25.8%)	11(11.5%)		
**Multisexual partner**	-	-	-	-	0.059	
No	0(0.0%)	4(7.7%)	3(9.7%)	7(7.3%)		
Yes	2(15.4%)	0(0.0%)	0(0.0%)	2(2.1%)		
Not mentioned	11(84.6%)	48(92.3%)	28(90.3%)	87(90.6%)		
**HTN (%)**	0(0.0%)	5(9.6%)	10(32.3%)	15(15.6%)	0.004*	
**DM (%)**	0(0.0%)	6(11.5%)	8(25.8%)	14(14.6%)	0.039*	
**Smoking**	-	-	-	-	0.142	
No	9(69.2%)	32(61.5%)	25(80.6%)	66(68.8%)		
Yes	3(23.1%)	15(28.8%)	2(6.5%)	20(20.8%)		
Not mentioned	1(7.7%)	5(9.6%)	4(12.9%)	10(10.4%)		
**History of GYN surgery (%)**	3(23.1%)	21(40.4%)	18(58.1%)	42(43.8%)	0.060	
**History of abdominal surgery (%)**	2(15.4%)	4(7.7%)	2(6.5%)	8(8.3%)	0.479	
**Pelvic organ prolapse (%)**	0(0.0%)	0(0.0%)	1(3.2%)	1(1.0%)	0.458	
**GYN structure problem (%)**	0(0.0%)	10(19.2%)	7(22.6%)	17(17.7%)	0.200	
**GI problem (%)**	0(0.0%)	1(1.9%)	0(0.0%)	1(1.0%)	1.000	
**Source**	-	-	-	-	0.178	
**OPD**	7(53.8%)	35(67.3%)	25(80.6%)	67(69.8%)		
**Ward**	6(46.2%)	17(32.7%)	6(19.4%)	29(30.2%)		
**Operation (%)**	0(0.0%)	5(9.6%)	1(3.2%)	6(6.3%)	0.495	

Data are presented as n (%) or mean ± standard deviation.

*p-value<0.05 was considered statistically significant after test.

BMI: body mass index, PID: pelvic inflammatory disease, UTI: urinary tract infection, IUD, intrauterine device, HTN: hypertension, DM: diabetes mellitus, GI: gastrointestinal, GYN: gynecologic, NA: not available, NSD: normal spontaneous delivery, C/S: cesarean section.

Table 2 shows the numbers and percentages of different categorical pathogens in PID patients among the three groups. There were 318 isolated microorganisms. There were six subgroups of pathogens, including vaginal, respiratory, enteric, skin, oral, and opportunistic human infections. The most common pathogens in PID patients in the age ≥45y group than in the other two groups were respiratory and other pathogens (p = 0.046 and p = 0.039, respectively). The vagina, enteric, skin, and oral pathogens were no significant differences among the three groups.

**Table 2:** Bacterial isolates in patients with pelvic inflammatory disease (n = 96).

	<25 y/o (A)	25–44 y/o (B)	≧45 y/o (C)	Total	P-value	Post-hoc
N	13	52	31	96		
**No. of infected organ type**	1.69±0.95	1.83±0.79	1.94±1.12	1.84±0.92	0.718	
Organism	-	-	-	-	-	
Vagina (%)	8(61.5%)	33(63.5%)	14(45.2%)	55(57.3%)	0.250	
Respiratory (%)	3(23.1%)	13(25.0%)	14(45.2%)	30(31.3%)	0.126	
Enteric (%)	8(61.5%)	39(75.0%)	19(61.3%)	66(68.8%)	0.356	
Skin (%)	1(7.7%)	3(5.8%)	5(16.1%)	9(9.4%)	0.254	
Oral (%)	2(15.4%)	7(13.5%)	5(16.1%)	14(14.6%)	0.923	
Other (%)	0(0.0%)	0(0.0%)	3(9.7%)	3(3.1%)	0.079	
**No. of organism isolates**	-	-	-	-	-	
Vagina	0.92±0.95	0.94±0.90	0.74±1.34	0.88±1.06	0.700	
Respiratory	0.23±0.44	0.29±0.54	0.65±0.92	0.40±0.69	0.046*	A<C
Enteric	0.69±0.63	1.15±0.92	1.29±1.85	1.14±1.27	0.362	
Skin	0.08±0.28	0.06±0.24	0.16±0.37	0.09±0.29	0.293	
Oral	0.23±0.60	0.17±0.47	0.16±0.37	0.18±0.46	0.898	
Other	0.00±0.00	0.00±0.00	0.10±0.30	0.03±0.18	0.039*	A<C
**No. of total bacteria type**	2.15±1.21	2.62±1.55	3.10±3.96	2.71±2.55	0.502	

Data are presented as n(%) or mean ± standard deviation.

*p-value<0.05 was considered statistically significant after test.

y/o: year-old.

Table 3 shows the number and percentage of different categorial pathogens in the PID patients. The *Prevotella bivia* (vaginal pathogens) was more common in age <25 and 25–44 years groups than in the age ≥45 years group (p = 0.035). The distribution *Klebsiella pneumonia* was not different among different age groups (p = 0.058).

**Table 3:** The numbers (percentage) of different categorial pathogens in pelvic inflammatory disease patients (n = 96).

	<25 y/o (A)	25–44 y/o (B)	≧45 y/o (C)	Total	P-value
N	13	52	31	96	
**Vagina (10 types)**	-	-	-	-	-
*Bacteroid thetaiotaomicron*	0(0.0%)	1(1.9%)	1(3.2%)	2(2.1%)	1.000
*Bacteroid uniformis*	0(0.0%)	0(0.0%)	1(3.2%)	1(1.0%)	0.458
*Bacteroides stercoris*	0(0.0%)	0(0.0%)	1(3.2%)	1(1.0%)	0.458
*Bacteroides vulgatus*	0(0.0%)	0(0.0%)	2(6.5%)	2(2.1%)	0.207
*Clostridium ramosum*	0(0.0%)	0(0.0%)	1(3.2%)	1(1.0%)	0.458
*Peptostreptococcus anaerobius*	3(23.1%)	14(26.9%)	7(22.6%)	24(25.0%)	0.894
*Peptostreptococcus species*	5(38.5%)	19(36.5%)	7(22.6%)	31(32.3%)	0.369
*Prevotella bivia*	3(23.1%)	15(28.8%)	2(6.5%)	20(20.8%)	0.035*
*Prevotella denticola*	0(0.0%)	0(0.0%)	1(3.2%)	1(1.0%)	0.458
*Staphylococcus saprophyticus*	1(7.7%)	0(0.0%)	0(0.0%)	1(1.0%)	0.135
**Respiratory (12 types)**	-	-	-	-	-
*Acinetobacter baumannii (CRAB)*	0(0.0%)	0(0.0%)	1(3.2%)	1(1.0%)	0.458
*Acinetobacter baumannii*	0(0.0%)	2(3.8%)	0(0.0%)	2(2.1%)	0.646
*Acinetobacter calcoaceticus complex*	0(0.0%)	0(0.0%)	0(0.0%)	0(0.0%)	1.000
*Haemophilus parainfluenzae*	0(0.0%)	2(3.8%)	0(0.0%)	2(2.1%)	0.646
*Klebsiella pneumonia*	1(7.7%)	4(7.7%)	8(25.8%)	13(13.5%)	0.058
*Prevotella melaninogenica*	1(7.7%)	1(1.9%)	1(3.2%)	3(3.1%)	0.388
*Pseudomonas aeruginosa*	0(0.0%)	1(1.9%)	2(6.5%)	3(3.1%)	0.712
*Serratia marcescens*	0(0.0%)	0(0.0%)	2(6.5%)	2(2.1%)	0.207
*Staphylococcus aureus*	1(7.7%)	3(5.8%)	3(9.7%)	7(7.3%)	0.863
*Staphylococcus aureus (ORSA)*	0(0.0%)	2(3.8%)	2(6.5%)	4(4.2%)	0.794
*Stenotrophomonas maltophilia*	0(0.0%)	0(0.0%)	1(3.2%)	1(1.0%)	0.458
*β-streptococci group A*	0(0.0%)	0(0.0%)	0(0.0%)	0(0.0%)	1.000
**Enteric (18 types)**	-	-	-	-	-
*Aeromonas hydrophila*	0(0.0%)	0(0.0%)	1(3.2%)	1(1.0%)	0.458
*Aeromonas sobria*	0(0.0%)	0(0.0%)	1(3.2%)	1(1.0%)	0.458
*Bacteroid fragilis*	0(0.0%)	5(9.6%)	5(16.1%)	10(10.4%)	0.360
*Bifidobacterium sp*.	0(0.0%)	0(0.0%)	2(6.5%)	2(2.1%)	0.207
*Citrobacter freundii*	0(0.0%)	2(3.8%)	0(0.0%)	2(2.1%)	0.646
*Enterobacter aerogenes*	0(0.0%)	1(1.9%)	0(0.0%)	1(1.0%)	1.000
*Enterococcus avium*	0(0.0%)	0(0.0%)	1(3.2%)	1(1.0%)	0.458
*Enterococcus faecalis*	2(15.4%)	8(15.4%)	4(12.9%)	14(14.6%)	1.000
*Enterococcus faecium*	0(0.0%)	2(3.8%)	1(3.2%)	3(3.1%)	1.000
*Enterococcus*	0(0.0%)	8(15.4%)	3(9.7%)	11(11.5%)	0.364
*Escherichia coli*	4(30.8%)	16(30.8%)	14(45.2%)	34(35.4%)	0.387
*Morganella morganii*	0(0.0%)	1(1.9%)	2(6.5%)	3(3.1%)	0.712
*Prevotella species*	0(0.0%)	0(0.0%)	0(0.0%)	0(0.0%)	1.000
*Proteus mirabilis*	0(0.0%)	0(0.0%)	3(9.7%)	3(3.1%)	0.079
*Streptococcus bovis II*	1(7.7%)	2(3.8%)	1(3.2%)	4(4.2%)	0.608
*Streptococcus group D non enterococcus*	0(0.0%)	1(1.9%)	0(0.0%)	1(1.0%)	1.000
*Veillonella species*	1(7.7%)	5(9.6%)	0(0.0%)	6(6.3%)	0.170
*β-streptococci group B*	1(7.7%)	9(17.3%)	2(6.5%)	12(12.5%)	0.323
**Skin (1 type)**	-	-	-	-	-
*Coagulase-negative staphylococc*i	1(7.7%)	3(5.8%)	5(16.1%)	9(9.4%)	0.254
**Oral (9 types)**	-	-	-	-	-
*Fusobacterium nucleatum*	0(0.0%)	0(0.0%)	1(3.2%)	1(1.0%)	0.458
*Fusobacterium species*	1(7.7%)	0(0.0%)	0(0.0%)	1(1.0%)	0.135
*Prevotella disiens*	0(0.0%)	1(1.9%)	0(0.0%)	1(1.0%)	1.000
*Prevotella intermedia*	0(0.0%)	2(3.8%)	0(0.0%)	2(2.1%)	0.646
*Prevotella oralis*	1(7.7%)	0(0.0%)	1(3.2%)	2(2.1%)	0.105
*Streptococcus anginosus*	0(0.0%)	1(1.9%)	0(0.0%)	1(1.0%)	1.000
*Streptococcus sanguis*	0(0.0%)	1(1.9%)	0(0.0%)	1(1.0%)	1.000
*Viridans streptococci*	1(7.7%)	2(3.8%)	3(9.7%)	6(6.3%)	0.408
*β-streptococci group G*	0(0.0%)	2(3.8%)	0(0.0%)	2(2.1%)	0.646
**Others (3 types)**	-	-	-	-	-
*Pantoea species*	0(0.0%)	0(0.0%)	1(3.2%)	1(1.0%)	0.458
*Pasteurella species*	0(0.0%)	0(0.0%)	1(3.2%)	1(1.0%)	0.458
*Shewanella putrefaciens*	0(0.0%)	0(0.0%)	1(3.2%)	1(1.0%)	0.458

Data are presented as n(%).

*p-value<0.05 was considered statistically significant after test.

Table 4 shows the factors associated with vagina, respiratory, and enteric pathogens infections. After multivariate analysis, we could not find the factors associated with these pathogens’ infections.

**Table 4:** Factors associated with vagina/respiratory/enteric infection (n = 96).

	Vagina		Respiratory		Enteric	
	OR (95% CI)	P-value	OR (95% CI)	P-value	OR (95% CI)	P-value
**Age group**	-	-	-	-	-	-
<25 y/o	Reference	NA	Reference	NA	Reference	NA
24–44 y/o	0.53(0.01, 23.53)	0.740	1.33(0.03, 51.88)	0.878	1.04(0.03, 40.76)	0.984
≧45 y/o	3.03(0.04, 223.81)	0.613	1.99(0.04, 109.71)	0.736	0.11(0.00, 12.21)	0.362
**BMI group**	-	-	-	-	-	-
Normal	Reference	NA	Reference	NA	Reference	NA
Underweight	0.00(NA)	1.000	0.00(NA)	1.000	1.19E10(NA)	1.000
Overweight	1.73(0.25, 12.05)	0.580	2.11(0.32, 14.06)	0.439	1.33(0.10, 17.96)	0.831
Obese	0.21(0.01, 2.95)	0.246	6.51(0.36, 116.50)	0.203	0.00(NA)	0.997
**Education**	-	-	-	-	-	-
Junior high school or under	Reference	NA	Reference	NA	Reference	NA
Senior high school	9.11(0.70, 118.39)	0.091	0.12(0.01, 1.63)	0.112	0.35(0.01, 21.34)	0.619
College or above	2.54(0.18, 35.57)	0.49	0.17(0.01, 3.30)	0.242	0.12(0.00, 9.64)	0.339
**Multipara**	-	-	-	-	-	-
No	Reference	NA	Reference	NA	Reference	NA
Yes	2.73(0.24, 30.81)	0.416	0.14(0.01, 3.36)	0.223	3.12(0.22, 44.67)	0.402
**Menopause**	-	-	-	-	-	-
No	Reference	NA	Reference	NA	Reference	NA
Yes	0.2(0.01, 4.02)	0.296	7.26(0.46, 113.34)	0.157	8.86E31(NA)	0.996
**Previous PID**	-	-	-	-	-	-
No	Reference	NA	Reference	NA	Reference	NA
Yes	1.24(0.19, 7.86)	0.822	0.77(0.10, 5.67)	0.796	0.82(0.04, 15.80)	0.898
**Associated UTI**	-	-	-	-	-	-
No	Reference	NA	Reference	NA	Reference	NA
Yes	7.73(0.77, 77.49)	0.082	0.24(0.03, 2.31)	0.218	0.09(0.00, 2.15)	0.136
**HTN**	-	-	-	-	-	-
No	Reference	NA	Reference	NA	Reference	NA
Yes	2.94(0.23, 36.9)	0.403	0.07(0, 1.45)	0.086	4.01(NA)	1
**DM**	-	-	-	-	-	-
No	Reference	NA	Reference	NA	Reference	NA
Yes	2.26(0.16, 32.32)	0.548	0.71(0.04, 12.12)	0.815	0.00(NA)	0.997
**Smoking**	-	-	-	-	-	-
No	Reference	NA	Reference	NA	Reference	NA
Yes	10.11(0.72, 141.13)	0.085	1.03(0.10, 10.56)	0.980	1.78E24(NA)	0.996
**History of GYN surgery**	-	-	-	-	-	-
No	Reference	NA	Reference	NA	Reference	NA
Yes	0.79(0.12, 5.21)	0.803	3.90(0.39, 39.14)	0.247	0.21(0.01, 7.42)	0.391
**History of abdominal surgery**	-	-	-	-	-	-
No	Reference	NA	Reference	NA	Reference	NA
Yes	0.69(0.04, 11.02)	0.794	1.35(0.08, 23.96)	0.837	6.22E7(NA)	0.998
**GYN structure problem**	-	-	-	-	-	-
No	Reference	NA	Reference	NA	Reference	NA
Yes	0.9(0.11, 7.45)	0.923	0.13(0.01, 2.58)	0.180	5.09(0.19, 135.87)	0.332

Data are presented as OR (95% CI).

*p-value<0.05 was considered statistically significant after test.

BMI: body mass index, PID: pelvic inflammatory disease, UTI: urinary tract infection, HTN: hypertension, DM: diabetes mellitus, GYN: gynecologic, NA: not available.

## Discussion

Our study reviewed the medical records of 96 patients (31, 52, and 13 in the age ≥45y group, 25–44, and <25 years groups, respectively). We found isolated respiratory and other pathogens were more common in the age ≥45y group than the other two groups. The vagna and enteric pathogens were the most common among all groups. We found *Prevotella bivia* (vaginal pathogens) was more common in age <25 and 25–44 years groups than in the age ≥45 years group. However, we could not find the association between age and microorganisms of different sources.

Eighty-five percent of PID cases are caused by sexually transmitted pathogens or bacterial vaginosis-associated pathogens [14]. Fewer than 50% of PID cases are now attributable to *N*. *gonorrhoeae* or *C*. *trachomatis* [6, 7]. By contrast, fewer than 15% of acute PID cases are not sexually transmitted and instead are associated with enteric or respiratory pathogens [15]. In our study, the distribution of microorganisms differed among the three groups. The percentage of pathogens from different origins were not different among the three groups. For isolated organisms, the respiratory and other pathogens were more in the age ≥45y group.

Ness et al. examined 1140 patients aged 13–36 and found that vaginal and enteric pathogens are the most common non-sexually transmitted bacterial pathogens in younger patients [16]. The pathogenesis of PID in young patients is generally associated with high sexual activity; therefore, the causal organisms are vaginal and enteric pathogens. However, in our study, we found that both vagina and enteric pathogens were the most common pathogens among all groups.

Postmenopausal patients had lower sexual activity and a smaller endocervical canal, thus decreasing the ability of *C*. *trachomatis* and *N*. *gonorrhoeae* to attach [17]. One study also showed a 5-fold decrease of PID caused by *C*. *Trachomatis* with age (age 16–19 years compared to age 35–44 years), so it is important to evaluate the other causes of PID in older women [11]. The pathogenesis of PID in these patients is more likely direct extension from adjacent intra-abdominal viscera, such as appendicitis, diverticulitis, and bowel perforation, rather than ascending infection. Structural abnormalities such as fluid accumulation due to myoma, polyps, or malignancy such as squamous cell cervical carcinoma are also associated with an increased risk of developing PID in patients aged ≥45 years [17]. Because of the potential for direct intra-abdominal infection, *E*. *coli* and *Klebsiella* are most frequently encountered rather than sexually transmitted pathogens in older patients [17]. Another study showed that bacterial vaginosis and trichomonas vaginalis were the most common pathogens responsible for PID among postmenopausal women [1]. About 11.5% of postmenopausal women are diagnosed with PID [1]. Our study makes up a deficiency in studying PID in older women. Respiratory and other pathogens were more isolated in the age ≥ 45 years group than the other two groups.

Other risk factors also play roles in PID. One study found that increasing parity, multiple sexual contacts, and associated pelvic organ prolapse are risk factors of PID [1]. However, in their patient population, there was no significant association with chronic illnesses, smoking, hypertension, DM, or UTI [1]. In our study, the common characteristics of the age ≥ 45 years group were the number of NSD, hypertension, DM, and menopause.

According to the CDC 2021 STD Treatment Guidelines for PID, empirical antibiotics with ceftriaxone and doxycycline are advised to treat infection of sexually transmitted pathogens [12]. However, routine treatment for aerobic pathogens and other pathogens remains controversial [12]. The current recommendation for the management of PID is focused on STD-related pathogens in younger patients. Our results showed respiratory pathogens play an important role among age ≥ 45 years women with PID which can help guide a more specific choice of antibiotic.

*Prevotella bivia* is anaerobic, gram-negative, bile-sensitive rods belonging to genus *Bacteroides* [18]. It may harbor metronidazole-resistant characteristics [19]. *Nim* gene is responsible for the resistance [20]. In our study, we found *Prevotella bivia* was more isolated pathogens in the age <25 years and 25–44 years group than in the age ≥ 45 years group. The antibiotics regimen contained metronidazole should be further evaluated for the younger age group.

*Klebsiella pneumonia* is a gram-negative bacteria found in human gut flora and causes pneumonia and genito-urinary infection commonly [21]. Most *klebsiella* are resistant to ampicillin [22]. *K*. *pneumonia* can cause a severe infection, such as meningitis or brain abscess, and can be life-threatening which needs advanced antibiotics treatment [23]. The previous study explored endocervical culture from 100 women and they found *Klebsiella pneumonia* occupied 2% of the study women [24]. The other study showed the prevalence of *Klebsiella pneumonia* infection in PID was 18.3% [25]. They also found the isolated *K*. *pneumonia* numbers were 24, 15, 10, 5 in the age 21–30, 31–40, 41–50 and >50 years group, respectively [25]. There seemed no difference among different age groups [25]. In our study, the distribution of *K*. *pneumonia* was also no different among different age groups.

This study has several limitations. First, this is a retrospective study that may have some data collection bias, and the sample size is small. Second, common pathogens such as *N*. *gonorrhoeae* and *C*. *trachomatis* were not included in our study because special culture media should be used. Last, our study was based on a single center in eastern Taiwan and therefore our results may not be generalizable to other populations.

In conclusion, the most common non-STD-related pathogens in our study were enteric and vaginal pathogens among all aged patients. Respiratory pathogens were significantly more common in the age ≥ 45 years group than other two groups. As current guidelines for PID treatment focus on younger patients and sexually transmitted pathogens, our study might be a reference for antibiotic choice in the broader population. More comprehensive studies should be done and further outcomes should be examined, particularly after antibiotic treatment.
